# Ultrasonic Extraction Process of Polysaccharides from *Dendrobium nobile* Lindl.: Optimization, Physicochemical Properties and Anti-Inflammatory Activity

**DOI:** 10.3390/foods11192957

**Published:** 2022-09-21

**Authors:** Hang Chen, Xueqin Shi, Lin Zhang, Li Yao, Lanyan Cen, Lian Li, Yiyi Lv, Chaoyang Wei

**Affiliations:** 1 Key Laboratory of Fermentation Engineering and Biological Pharmacy of Guizhou Province, School of Liquor and Food Engineering, Guizhou University, Guiyang 550025, China; 2 Key Laboratory of Plant Resource Conservation and Germplasm Innovation in Mountainous Region (Ministry of Education), Institute of Agro-Bioengineering, College of Life Sciences, Guizhou University, Guiyang 550025, China

**Keywords:** *Dendrobium nobile* Lindl., polysaccharides, ultrasonic extraction, optimization, anti-inflammatory activity

## Abstract

To optimize the ultrasonic extraction process of polysaccharides from *Dendrobium nobile* Lindl. (DNP), the extraction method was conducted through a single-factor test and the response-surface methodology (RSM). With the optimal extraction process (liquid–solid ratio of 40 mL/g, ultrasonic time of 30 min, and ultrasonic power of 400 W), the maximum extraction yield was 5.16 ± 0.41%. DNP1 and DNP2 were then fractionated via DEAE-QFF and Sephacryl S-300 HR chromatography. The molecular weight (Mw) of DNP1 was identified as 67.72 kDa, composed of Man (75.86 ± 0.05%) and Glc (24.14 ± 0.05%), and the Mw of DNP2 was 37.45 kDa, composed of Man (72.32 ± 0.03%) and Glc (27.68 ± 0.03%). Anti-inflammatory assays results showed that as DNPs were 200 μg/mL, and the contents of NO, TNF-α, IL-1β, IL-6 and IL-10 in LPS-induced RAW 264.7 cells were about 13.39% and 13.39%, 43.88% and 43.51%, 17.80% and 15.37%, 13.84% and 20.66%, and 938.85% and 907.77% of those in control group, respectively. It was indicated that DNP1 and DNP2 inhibited the inflammatory response of RAW 264.7 cells induced by LPS via suppressing the level of NO and pro-inflammatory cytokines (TNF-α, IL-1β and IL-6) and promoting the secretion of anti-inflammatory cytokine (IL-10). Therefore, DNP1 and DNP2 have potential applications in the treatment of inflammatory injury.

## 1. Introduction

The valuable Chinese herb *Dendrobium nobile* Lindl. has been utilized extensively for thousands of years, mostly in the Chinese provinces of Guizhou, Yunnan, and Guangxi, as well as in subtropical areas south of the Yangtze river basin. [1]. According to recent investigations, *D. nobile* has been utilized to treat malignancies, hyperlipidemia, hyperglycemia, and even aging-related neurological diseases [2]. It is reported that *D. nobile* is rich in biologically active substances, including polysaccharides, alkaloids, bibenzyl, stilbene, glycosides, sesquiterpenes, fluorenone, and phenanthrene. Polysaccharides were the main active substances of *D. nobile*, and a key element with anti-aging, antioxidant, hypoglycemic, anti-cancer, and immune activity [3]. The polysaccharides of *D. nobile* have lately drawn more attention from scientists. Zhang et al. reported that JCP-40 derived from *D. nobile* was a straight-chain glucomannan (Mannose:glucose = 74.97%:25.03%) that consisted of β-1,4-ᴅ-Man*p*, and β-1,4-ᴅ-Glc*p* residues, and some of acetyl groups were linked to the C-2 or C-3 positions of the mannose residues [4].

Growing evidence suggests that the structure properties of polysaccharides, for instance, their chemical structure, Mw, and monosaccharide composition, can be affected by various extraction methods, which are related to their biological activity [5,6]. To prepare polysaccharides, it is required to choose an appropriate extraction technique. Modern extraction techniques include supercritical fluid extraction, microwave extraction, pressurized liquid extraction, ultrasonic extraction, and enzyme-assisted extraction, and solvents include supercritical fluids, ionic liquids, and solvent-free conditions [7,8]. However, all of these extraction methods have some disadvantages, including their high cost, high pressure, laborious preparation, and a lack of knowledge regarding the harm caused by substitutive organic solvents. It is reported that ultrasonic extraction is a mild and environmentally friendly method [9,10]. By increasing temperature and pressure through cavitation processes, ultrasound technology facilitates the solubility, diffusivity, and transport of solute molecules. Additionally, cavitation can mechanically erode solid particles and produce tiny bubbles that make it easier for cells to release polysaccharides. Cavitation can also enhance the contact between target compound and solvent, facilitating higher solvent penetration into the sample matrix via mechanical effects [11]. These effects result in reducing the waste of the solvent, speeding up extraction time, and increasing polysaccharide extraction yield [12]. Furthermore, RSM (response-surface methodology) is often used in industry and laboratories for process optimization through the use of different statistical techniques. The response-surface method fits the correspondence between variables and response values through equations, and solves equations to find the optimal combination of parameters for the purpose of process optimization [13]. Mzoughi [14] et al. used Box–Behnken design (BBD) to optimize the extraction rate of *Suaeda fruticosa* polysaccharides. The three independent variables of temperature, time, and pH were investigated and the optimal settings were 90 °C, 37 min, and 2.9. Under these conditions, the separation yield was 34.0%.

Extraction of polysaccharides is a key step in their development and application. Therefore, an effective extraction method must be investigated, and the extraction process must be optimized to obtain high yields of DNPs. It has been shown that ultrasonic extraction is an effective extraction method and can moderately degrade polysaccharides to enhance their biological activity [9]. However, studies on the ultrasonic water-extraction process of DNPs are rare. The extraction and purification of DNPs have not received sufficient attention, leading to the loss and waste of DNPs. The aim of this work was to optimize the ultrasonic extraction process of DNP via a single factor test and RSM. Additionally, DNPs’ physicochemical characteristics and anti-inflammatory activity were investigated. The results of the present work will provide a scientific basis for the in-depth study and application of the extraction process, structure, and anti-inflammatory activity of DNPs.

## 2. Materials and Methods

### 2.1. Materials and Reagents

*Dendrobium nobile* Lindl. was purchased from Guizhou Chishui Guo Li *Dendrobium* Development Co., Ltd. (Chishui, China). After drying and crushing, it was sieved through 80 meshes and left on standby. Ten kinds of standard monosaccharides, such as mannose, ribose, rhamnose, glucuronic acid, galacturonic acid, glucose, galactose, xylose, arabinose, and fucose, were purchased from Aladdin Reagent Co., Ltd. (Shanghai, China). ELISA kits (including TNF-α, IL-1β, IL-6 and IL-10) were purchased from Shanghai Jianglai Biological Co., Ltd. (Shanghai, China). DEAE-Sepharose Fast Flow (DEAE-QFF) and Sephacryl S-300 HR were purchased from GE Healthcare. Other chemicals and reagents were of analytical grade.

### 2.2. Optimal Design of Ultrasonic Extraction Process for DNP

#### 2.2.1. Ultrasonic Extraction Procedure

*D. nobile* polysaccharides were extracted in an ultrasonic homogenizer (LC-JY92-IIN, Shanghai Lichen Technology Co., Ltd., Shanghai, China). The initial ultrasonic extraction procedure was as follow: 1.0 g of *D. nobile* powders were extracted under distilled water with a liquid–solid ratio of 40 mL/g, ultrasonic power of 300 W, and ultrasonic time of 20 min at 25 °C. The supernatant was obtained by centrifuging at 8000 r/min for 15 min, then adequately mixed with a fourfold volume of anhydrous ethanol at room temperature overnight. The precipitate was dried to a constant weight at 60 °C after centrifugation at 8000 r/min for 15 min. The yield of DNP was measured by weighing. The calculation formula was as follows:

$$Extraction\;yield(\%,\;\frac{w}{w})=\frac{m}{M}\times 100\%,$$

where *m* and *M* were the mass of polysaccharides (g) and *D. nobile* powders (g), respectively.

#### 2.2.2. Single Factor Design

The influence of three factors on extraction yield including a liquid–solid ratio of 20, 30, 40, 50, and 60 mL/g; ultrasonic time of 5, 10, 20, 30, and 40 min; and ultrasonic power of 100, 200, 300, 400, and 500 W were investigated.

#### 2.2.3. RMS Optimization Design

Based on the preliminary screening of extraction factors (liquid–solid ratio, ultrasonic time, and ultrasonic power) in the single-factor trials, Box-Behnken Design (BBD) was used to optimize the parameters via Design-Expert 8.0.6.1 software (Stat-Ease Inc., Minneapolis, MN, USA). The independent variables and levels of the experiments are listed in Table 1.

### 2.3. Isolation and Purification of DNP

Deproteinization of DNP was conducted by the enzymatic method combined with the Sevag method [15]. Firstly, papain and 10 mg/mL of DNP were mixed in a 1:10 (m/m) ratio. The mixture was boiled in boiling water for 10 min to inactivate the papain after being placed in the water bath at 55 °C for 2.5 h. Subsequently, 1/5 volume of Sevag reagent (chloroform: *n*-butanol = 4:1) was added to the mixture, and it was shaken vigorously for 30 min. The aqueous phase was continued to de-protein after centrifugation (8000 r/min, 10 min). This was repeated 5 times until no protein suspension was observed. Finally, the aqueous phase was transferred to a rotary evaporator to remove the residual organic phase under reduced pressure, and freeze-dried at −60 °C, 0.01 Mpa for 48 h.

The deproteinized DNP (10 mg/mL) was dissolved in distilled water and loaded onto a DEAE-QFF (30 × 5 cm) column. The eluent was distilled water, flowing at a rate of 3 mL/min. The eluent was collected and concentrated before being freeze-dried. The lyophilized polysaccharide was then separated at 10 mg/mL on the HiPrep Sephacryl S-300 HR (GE Healthcare, Sweden) by eluting with 0.15 M PBS (pH 7.2). 0.5 mL/min was the flow rate. The elution was collected in 10 mL tubes and monitored at 490 nm using the phenol-sulfuric acid method [16]. The eluate from the same fraction was combined, concentrated, dialyzed (MWCO = 3.5 kDa), and freeze-dried.

### 2.4. Structural Characterization of DNPs

#### 2.4.1. Measurement of Monosaccharide Composition and Mw of DNPs

The pre-column derivatization method was used to detect the monosaccharide composition of DNP [17]. A SEC-MALLS-RI instrument was applied to measure the Mw of DNPs, which includes a laser photometer (DAWN HELEOS-II, Wyatt Technology Co., Santa Barbara, CA, USA) combined with three columns (Shodex OH-pak SB-805, 804, and 803, 300 × 8 mm, Showa Denko K.K., Tokyo, Japan) in series and an RI (differential refractive index) detector (Optilab T-rEX, Wyatt Technology Co., Santa Barbara, CA, USA). The column temperature was held at 45 °C, and 1 mg/mL of DNPs was dissolved in the mobile phase (0.1 M NaNO_3_ aqueous solution containing 0.02% NaN_3_), and filtered through a filter of 0.22 μm. A total of 0.141 mL/g was determined to be the *dn/dc* value of DNPs. The flow rate was 0.4 mL/min.

#### 2.4.2. Spectra Analysis of DNPs

DNP functional groups were determined by Fourier infrared spectroscopy [18]. A 2 mg quantity of DNPs was ground and pressed with 40 mg KBr, scanned with a Fourier-transform infrared spectrometer (Frontier, PerkinElmer, Waltham, MA, USA) at 4000–400 cm^−1^ with a resolution of 4 cm^−1^. The UV-Vis spectrum of the DNPs was recorded using the UV-2700 ultraviolet spectrophotometer (Shimadzu, Japan) in the spectral scanning range of 200−400 nm^−1^. Primary structures of DNPs were analyzed using nuclear magnetic resonance (NMR) spectroscopy. In brief, 30 mg of polysaccharide was dried in a desiccator with P_2_O_5_ for 48 h, dissolved in 1 mL of D_2_O, and lyophilized. This was then repeated twice to exchange hydrogen protons. Finally, it was dissolved in 1 mL of D_2_O with acetone as an internal standard before being transferred to an NMR tube. Spectra of ^1^H and ^13^C NMR were recorded by a Bruker Advance 600 MHz spectrometer (Bruker, Rheinstetten, Germany) at 25 °C. Chemical shifts were expressed as ppm; acetone was internal standard (2.12/31.00 ppm for ^1^H/^13^C). Spectra processing was conducted in MestReNova 14.0.0-23239 (Mestrelab Research S.L. Inc., Santiago de Compostela, Spain) software.

### 2.5. Anti-Inflammatory Activity Assay

#### 2.5.1. Cell Culture

RAW 264.7 murine macrophage cells (purchased from Cell Resource Center, IBMS, CAMS/PUMC, Beijing, China) in logarithmic phase were collected, and their concentration of cell suspension was adjusted to 1 × 10^5^ cell/mL. To each well was added 100 μL of cell suspension in a 96-well plate, while the edge wells received sterile PBS instead. It was then incubated at 37 °C for 24 h in a cell incubator with 5% CO_2_. After cells were plastered, it was replaced with serum-free DMEM medium and LPS (1 mg/mL), and then incubated at 37 °C in the cell incubator overnight. A 100 µL quantity of the concentration gradient (200, 100, 50, 25, 12.5 μg/mL) of DNPs that were dissolved in the DMEM solution was added; the control group only replaced the DNP solution with DMEM, the blank group replaced cell suspension and the DNP solution with DMEM. There were three parallels for each group. The cells were cultured for 24 h at 37 °C in the cell incubator [19].

#### 2.5.2. Cell Proliferation and Cytotoxicity Assay

Cell proliferation and cytotoxic activity were detected by the CCK-8 method. The preparation of CCK-8 working solution was as follows: CCK-8 solution was mixed with serum-free DMEM medium at a volume ratio of 1:10. The cells were sucked off the old medium, washed twice with PBS, and CCK-8 mixture was added to cover each sample. After incubation at 37 °C for 3 h, 100 µL of the mixture was extracted and added to each well, and the microplate reader (Labsystems Multiskan MS 352, Vantaa, Finland) was performed to measure the absorbance at 450 nm [20].

#### 2.5.3. Determination of NO and Cytokine Content

The supernatant culture fluid was centrifuged at 1000 r/min for 10 min at 4 °C after incubation. The content of NO was determined using a commercial kit (Shanghai Jianlai Biotechnology Co., Ltd., Shanghai, China) and the cytokine contents (including TNF-a, IL-1β, IL-6, and IL-10) were detected by the Elisa kits (Shanghai Jianlai Biotechnology Co., Ltd., Shanghai, China) [16]. For the specific operations, refer to the instructions.

### 2.6. Statistical Analysis

Experiments were designed with three replications; the data are presented as mean ± standard deviation. The significant difference (*p* < 0.05) between the treatment groups was assessed by ANOVA and one-way Duncan’s test using SPSS 21.0 software (IBM Co., Chicago, IL, USA). OriginPro 9.0 (MicroCal Inc., MA, USA) was used for graphing.

## 3. Results and Discussion

### 3.1. Single Factor Experiments Assessment

**Figure 1:**
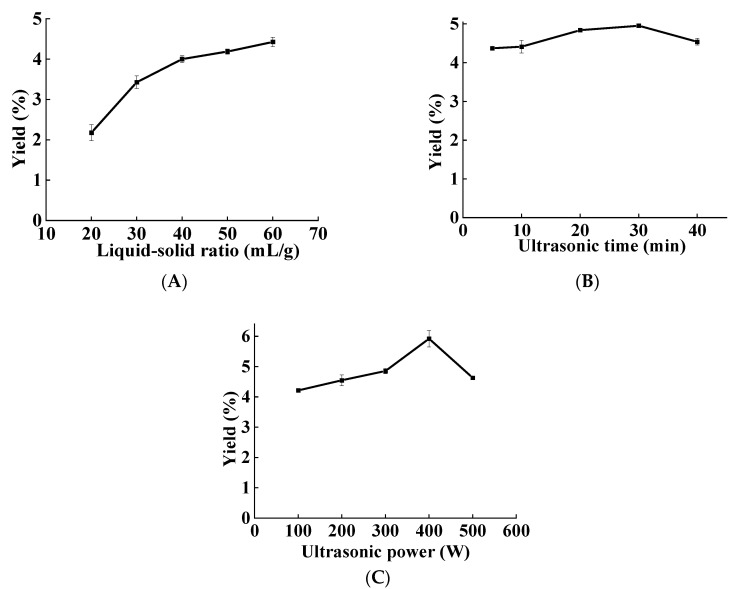
Effect of three factors on the yield of DNP, (**A**) liquid–solid ratio, (**B**) ultrasonic time, and (**C**) ultrasonic power.

Figure 1A revealed the effect of the liquid–solid ratio on the DNP yield, which exhibited an increasing trend at the range of 20–60 mL/g. The increasing trend of polysaccharide yield was very obvious before the liquid–solid ratio reached 40 mL/g, but slowed down after exceeding 40 mL/g. Moreover, excessive liquid–solid ratio would reduce the efficiency of ultrasound medium to absorb super energy and increase energy consumption. Consequently, 40 mL/g was the optimal liquid–solid ratio.

The ultrasonic time is a key parameter affects the DNP yield. It was increased with the extension of ultrasonic time and met the maximum at 30 min; however, it decreased when the ultrasonic time was extended further (Figure 1B). This might be due to the longer ultrasonic time destroying the structure of the polysaccharides and degrading them, which resulted in decreasing the extraction yield [21]. Therefore, the optimal ultrasonic time was 30 min.

Ultrasonic power affects polysaccharide yield by influencing solubility of the target compound in solvent and heat generation [22]. As shown in Figure 1C, as ultrasonic power was raised, the DNP yield steadily increased and peaked at 400 W. The DNP yield was then reduced, possibly because of excessive ultrasonic power degrading the polysaccharides [21] Therefore, the optimal ultrasonic extraction power of DNP was 400 W.

### 3.2. RSM Optimization of Ultrasonic Extraction

#### 3.2.1. Fitting the Model

Polysaccharide extraction takes place in a complicated environment [23]. Tan et al. [24] used RSM and an artificial neural network to optimize ultrasound-assisted dual aqueous phase extraction of *Cornus officinalis* polysaccharides. The yield of COPS was 7.85 ± 0.09% under the conditions of ultrasonic power of 350 W, extraction temperature of 51 °C, liquid-to-solid ratio of 17 mL/g, and extraction time of 38 min. Therefore, to further investigate the correlations between the elements and establish the ideal extraction conditions, the Box–Behnken design was employed. Based on the above results from the single factor experiment, a liquid–solid ratio of 30.00–50.00 mL/g, ultrasonic time of 20–40 min, and ultrasonic power of 300–500 W (Table 1) were employed out to further optimize the BBD experiments for DNP extraction. A total of 17 runs of the BBD experiment were conducted randomly, and the results are listed in Table 2.

On the basis of BBD results, the coded regression equation was obtained by fitting the three-factor results:

Y = 0.053 + 0.002X_1_ − 0.003X_2_ + 0.00006X_3_ − 0.004X_1_X_2_ + 0.001X_1_X_3_ − 0.002X_2_X_3_ − 0.011X_1_^2^ − 0.004X_2_^2^ − 0.009X_3_^2^

where X_1_, X_2_, and X_3_ represented the parameters of liquid–solid ratio, ultrasonic duration, and ultrasonic power, respectively, and Y represented the yield of DNP (%).

The statistical ANOVA results were listed in Table 3. The model was significant due to the high F-value (32.29) and low *p*-value (0.0001). The value of R^2^ (the determination coefficient), R^2^_adj_ (the adjusted determination coefficient), and CV (the coefficient of variation) in the proposed model were 0.9765, 0.9462, and 4.92%, respectively. The predicted values and measured values exhibited a strong correlation, demonstrating the model’s excellent reliability. Moreover, the *p*-value (0.9803) of the lack of fit and F-value (0.056) for the model suggested that it was not significant compared to the pure error. The polysaccharide yields were significantly impacted by the linear coefficients (X_1_ and X_2_), interaction-term coefficients (X_1_X_2_ and X_2_X_3_), and quadratic term coefficients (X_1_^2^, X_2_^2^, and X_3_^2^), in the following order of magnitude: ultrasonic time > liquid–solid ratio > ultrasonic power.

#### 3.2.2. Analysis of Response Surfaces

**Figure 2:**
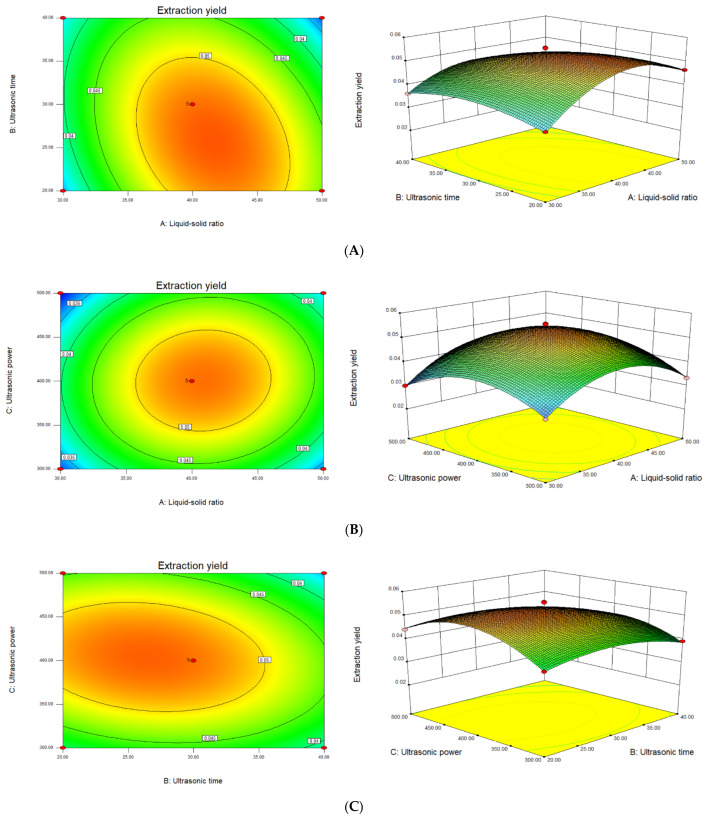
Contour plots and response-surface plots of the interactive effects of different factors on the DNP yield, (**A**) liquid–solid ratio and ultrasonic time, (**B**) liquid–solid ratio and ultrasonic power, and (**C**) ultrasonic time and ultrasonic power.

The impact of three dependent factors and their interaction on DNP extraction yield can be visualized and illustrated by combining 3D response-surface plots with contour plots. Table 3 and Figure 2 show the *p* value of X_1_X_2_ < 0.01, suggesting that the mutual interactions between liquid–solid ratio and ultrasonic time had a considerable impact on the DNP yield. The response value was increased with the increase in the liquid–solid ratio and ultrasonic time in the range of 20–40 mL/g and 20–30 min, but decreased when the two factors exceeded these ranges. The interaction of other factors had no significant effect on the DNP yield.

In summary, the optimal ultrasonic extraction process of DNP was as follows: a liquid–solid ratio of 40 mL/g, an ultrasonic time of 30 min, and an ultrasonic power of 400 W. Under these conditions, the maximum yield predicted by the model was 5.29%. The verification experiment demonstrated that the actual DNP yield was 5.16 ± 0.41% (*n* = 3), which was extremely near to the predicted value. It suggested that the model was effective for the extraction of DNP.

### 3.3. The Physicochemical Properties of DNPs

**Figure 3:**
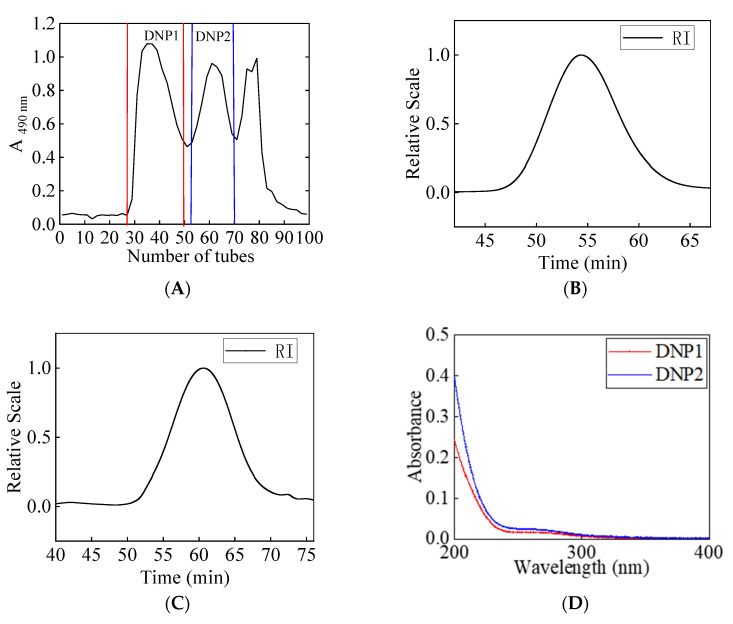

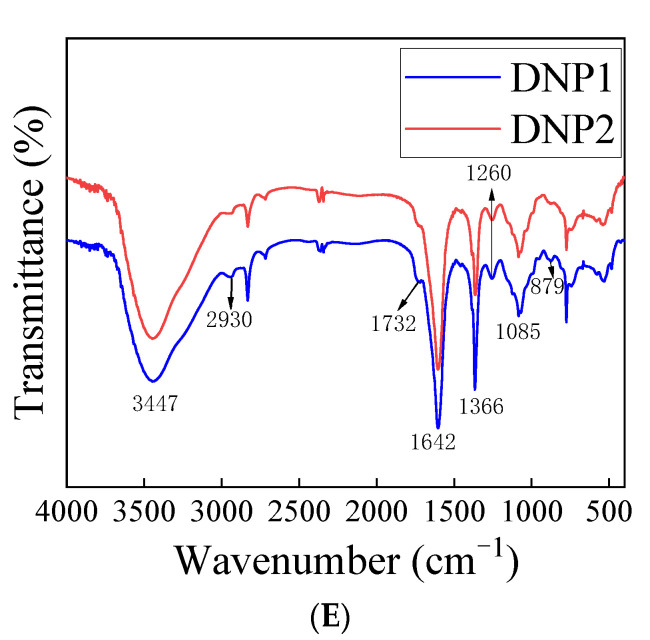
Elution curve of DNPs by Sephacryl S-300 HR (**A**), molecular weight distribution of DNP1 (**B**) and DNP2 (**C**), UV absorption spectrum (**D**), Fourier infrared spectroscopy (**E**).

Two fractions were obtained after DNP were separated and purified (Figure 3A). DNP1 and DNP2 were in the homogeneous fraction, which showed a symmetrical narrow peak on chromatograms (Figure 3B,C) with an Mw of 67.72 (±0.811%) kDa and 37.45 (±0.936%) kDa, respectively. In the UV absorption spectrum, DNP1 and DNP2 showed no absorption peaks at wavelengths 200–400 nm (Figure 3D), indicating that DNP1 and DNP2 did not contain proteins and nucleic acids. The results revealed that monosaccharide composition of DNP1 was primary composed of mannose (75.86 ± 0.05%) and glucose (24.14 ± 0.05%), and DNP2 was mainly composed of mannose (72.32 ± 0.03%) and glucose (27.68 ± 0.03%).

### 3.4. Structure Analysis of DNPs

Infrared absorption spectroscopy is a tool for analyzing the structure of polysaccharides, which can be identified based on the characteristic absorption peaks of their functional groups. Result showed that the FT-IR spectra of DNP1 and DNP2 were basically the same (Figure 3E). A strong absorption at 3447 cm^−1^ was assigned to the stretching vibration of O-H [25]. The signal at 2930 cm^−1^ could be associated with the C-H stretching vibration [26]. The peak near 1732 cm^−1^ was represented the acetyl group [27]. The band at 1642 cm^−1^ was attributed to the scissoring vibration of bound water [28]. The absorption peaks around 1366 cm^−1^ were due to the C-H deformation vibration [29]. The peaks around 1200–1000 cm^−1^ were attributed to C-O-H side groups and C-O-C glycosidic bond vibrations, suggesting the presence of pyran rings [30]. An absorption peak was found at 879 cm^−1^, representing β-configuration C-H bond [31].

**Figure 4:**
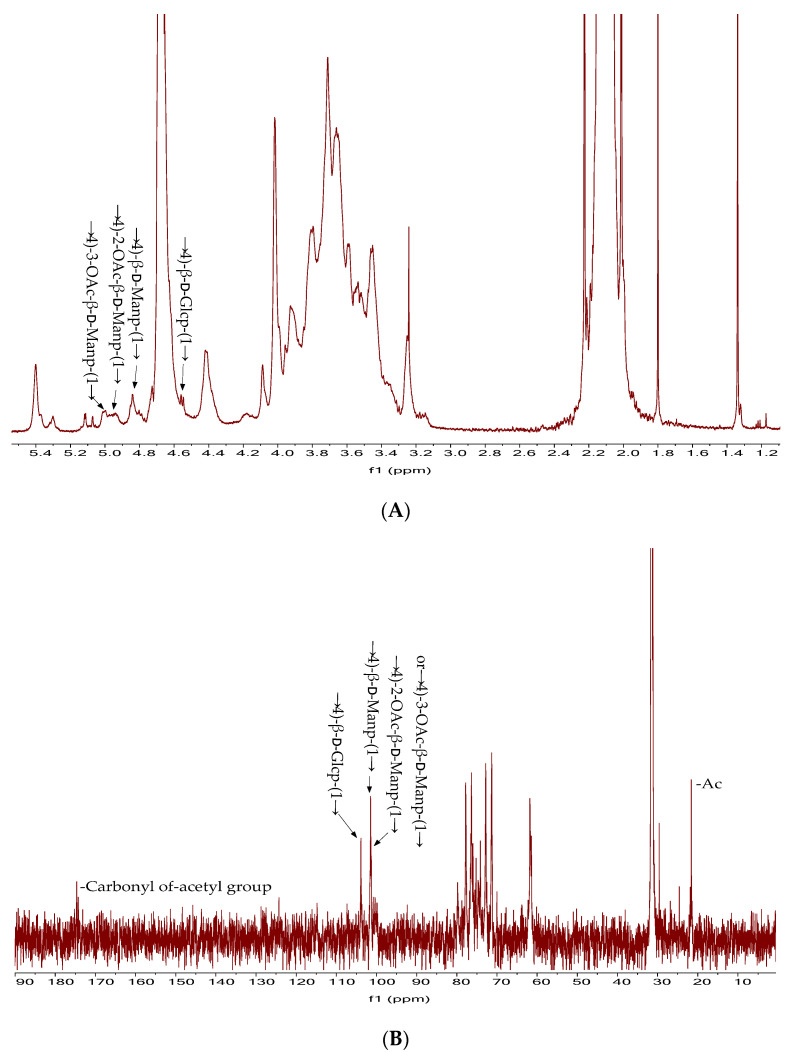
^1^H NMR spectrum (**A**); ^13^C NMR spectrum (**B**).

The monosaccharide composition and IR spectra of DNP1 and DNP2 were basically the same, and the content of DNP1 was greater than that of DNP2. Therefore, DNP1 was selected as representative for NMR analysis. As shown in Figure 4A, the strong chemical shift at δ 4.78 ppm in ^1^H’s NMR spectrum was the water peak of the D_2_O solvent [32]. The anomeric protons of β-glycoside were δ 4.4–5.0 ppm, and those of α-glycoside were δ 5.0–5.4 ppm [33]. There was a group signal at 4.50–5.50 ppm in ^1^H NMR, suggesting that DNP1 was a heteropolysaccharide containing multiple glycosidic residues. The signal at δ 4.4–5.0 ppm in ^1^H NMR (Figure 4A) and δ 100–105 ppm in the ^13^C NMR (Figure 4B) spectrum indicated that DNP1 was mainly composed of β-type linked glycosidic residues.

Four anomeric protons were observed in the ^1^H NMR spectrum at δ 4.99, δ 4.94, δ 4.83, and δ 4.56 ppm, which were assigned to β-1,4-3-OAc-ᴅ-Man*p*, β-1,4-2-Oac-ᴅ-Man*p*, β-1,4-ᴅ-Man*p*, and β-1,4-ᴅ-Glc*p*. In the ^13^C NMR spectrum of DNP1, δ 103.32, δ 100.90 and 100.74 ppm were attributed to β-1,4-ᴅ-Glc*p*, β-1,4-ᴅ-Man*p*, β-1,4-3-OAc-ᴅ-Man*p* or β-1,4-2-OAc-ᴅ-Man*p*. The carbon signals at δ 21.06 ppm and δ 174.08 ppm in ^13^C NMR spectrum were attributed to the carbonyl and methyl groups of the acetyl group, respectively [31]. In summary, according to monosaccharide composition, FT-IR spectra, and NMR data, combined with previous studies, DNP1 is a straight-chain glucomannan composed mainly of β-1,4-ᴅ-Man*p*, β-1,4-ᴅ-Glc*p* residues, and some acetyl groups linked to C-2 or C-3 of the mannose residues [4].

### 3.5. Anti-Inflammatory Activity

#### 3.5.1. Cell Proliferation and Cytotoxicity Assessment

**Figure 5:**
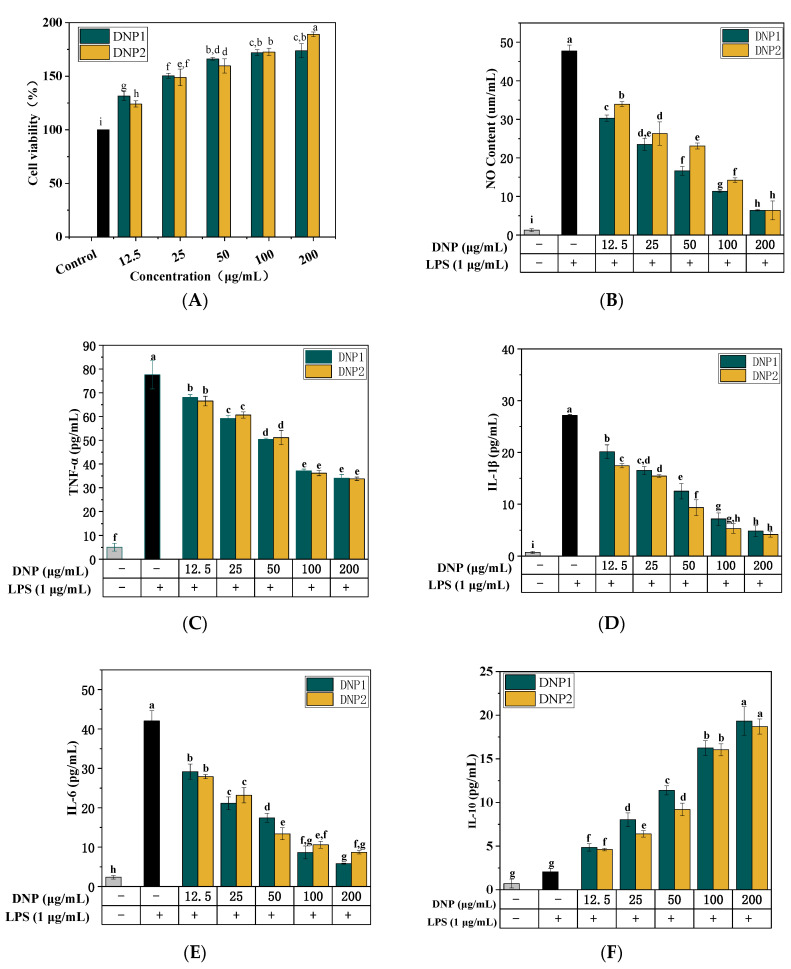
Effect of DNPs on the proliferation of RAW 264.7 macrophages (**A**); Effect of DNPs on the content of NO and cytokines by RAW 264.7 macrophages, (**B**) NO, (**C**) TNF-α, (**D**) IL-1β, (**E**) IL-6, and (**F**) IL-10. Different lowercase above the columns indicating significant differences among groups (*p* < 0.05).

Macrophages are important to host defense and innate immune response [19]. However, before studying their in vitro anti-inflammatory activities, the evaluation of DNP on the cell proliferative activities and cytotoxicity against RAW264.7 is necessary. As the results revealed, both DNP1 and DNP2 had cell proliferative effects on RAW264.7 and were concentration dependent (Figure 5A). Moreover, the proliferative effects increased with the rise of polysaccharide concentration. The cell viability when treated with DNP1 and DNP2 reached 173.73 ± 6.61% and 189.08 ± 2.12%, while the polysaccharide concentration reached 200 μg/mL. There were significant differences (*p* < 0.05) compared with the control group. It was indicated that DNP1 and DNP2 had a significant cell proliferation on RAW264.7 without cytotoxicity within setting polysaccharide concentration.

#### 3.5.2. Effect of DNPs on the Content of NO and Cytokines

Lipopolysaccharide (LPS) is commonly utilized as a natural immunological and inflammatory stimulant, and is an endotoxin derived from the outer membrane of Gram-negative bacteria [34]. It has been reported to trigger inflammatory responses by activating a series of intercellular signaling pathways, resulting in the release of pro-inflammatory cytokines in cells [35]. Therefore, in this study, LPS was used to induce RAW 264.7 cells to conduct the inflammatory tests, and the NO and ELISA kits were performed to assess the content of NO and cytokines.

NO is an important inflammatory substance that regulates the immune response [36]. We explored the anti-inflammatory action of DNPs, whose effect on the NO generation of macrophages was determined. As shown in Figure 5B, the NO content of RAW 264.7 rose considerably following incubation with LPS (1 μg/mL), reaching 47.71 ± 1.56 μg/mL. While treatment with different doses of DNP1 and DNP2, the NO produced by RAW 264.7 reduced significantly and showed a concentration dependence. When the concentration of DNP1 and DNP2 was 200 μg/mL, the NO content was 6.39 ± 0.18 μg/mL and 6.39 ± 2.44 μg/mL, respectively. At low concentration levels (12.5–100 μg/mL), the inhibitory effect of DNP1 on NO was significantly stronger than that of DNP2.

Additionally, activated RAW 264.7 cells secreted amounts of the tumor necrosis factor TNF-α in response to the tissue injury of inflammation [37]. TNF-α is a pro-inflammatory cytokine in charge of a wide range of intracellular signaling events, induces cell necrocytosis and apoptosis, and activates the release of cytokines, for instance, IL-6 and IL-1β [35]. Similar to TNF-α, two important immune cytokines (IL-6, IL-1β) are in charge of a string of intracellular signaling activities that affect monocytes and macrophages and improve immune function [38]. Nevertheless, the overexpression of cytokines is harmful to humans [39]. As illustrated in Figure 5C, the content of TNF-α was increased by 15.34-fold to 77.61 ± 5.95 pg/mL in response to stimulation of LPS. Compared with the control group, DNPs treatment inhibited LPS-induced RAW 264.7 to secrete TNF-α. The content of TNF-α in DNP1 and DNP2 treatment groups at range of 12.5–200 μg/mL was significantly lower than that of the control group. TNF-α production did not significantly change between DNP1 and DNP2.

Inflammatory mediator of IL-1β plays a role in the initiation and escalation of the inflammatory response that results in intestinal damage [40]. Figure 5D demonstrated that the IL-1β level in the control group reached 27.13 ± 0.20 pg/mL, which was 38.06 times of the blank group, while IL-1β level in DNPs groups were significantly inhibited. When the dose of DNPs was 200 μg/mL, the contents of IL-1β in DNP1 and DNP2 groups were 4.83 ± 1.03 pg/mL and 4.17 ± 0.50 pg/mL, respectively. They were greatly lower than those in the control group. Furthermore, the increase in IL-6 is considered to be a typical sign of acute inflammatory response [41]. LPS obviously stimulated IL-6 produced compared with the blank group. In contrast, the addition of DNPs significant (*p* < 0.05) suppressed IL-6 production in RAW 264.7 and exhibited a reliance on the concentration of DNPs (Figure 5E). DNPs fraction greatly increased the production of IL-10 and showed reliance on the concentration compared with the control group (Figure 5F). These results demonstrated that DNPs had anti-inflammatory activity.

## 4. Conclusions

In this study, *Dendrobium nobile* Lindl. was used as raw material to extract polysaccharides by ultrasonic method, and the extraction parameters were optimized using a single-factor test and an RSM design. The optimal extraction process involved a liquid–solid ratio of 40 mL/g, ultrasonic power of 400 W, ultrasonic time of 30 min, and had an extraction yield of 5.16 ± 0.41%. The crude polysaccharide was purified to obtain DNP1 and DNP2 fractions. DNP1 was an Mw of 67.72 kDa and composed of mannose (75.86 ± 0.05%) and glucose (24.14 ± 0.05%). DNP2 has an Mw of 37.45 kDa and was composed of mannose (72.32 ± 0.03%) and glucose (27.68 ± 0.03%). The spectra results showed that the structures of DNP1 and DNP2 were basically the same, indicating that the effect of ultrasonic extraction on DNPs was mainly on the change in molecular weight, and did not affect the primary structure. The results of anti-inflammatory assays showed that both DNP1 and DNP2 had anti-inflammatory activity without significant difference. The NO content of RAW 264.7 induced by LPS was 47.71 ± 1.56 μg/mL. Under treatment with DNP1 and DNP2 at a concentration of 200 μg/mL, the NO content was 6.39 ± 0.18 μg/mL and 6.39 ± 2.44 μg/mL, respectively. Similarly, the contents of TNF-α, IL-1β and IL-6 were 34.06 ± 1.61 and 33.77 ± 0.76 pg/mL, 4.83 ± 1.03 and 4.17 ± 0.50 pg/mL, and 5.82 ± 0.12 and 8.69 ± 0.43 pg/mL, which were significantly lower than the control group. The content of IL-10 was 19.34 ± 1.63 and 18.70 ± 0.86 pg/mL, which is significantly higher than the control group. These results suggested that DNPs may exert anti-inflammatory activity against LPS-induced RAW 264.7 cells by reducing the content of NO and pro-inflammatory cytokines (TNF-α, IL-1β, and IL-6) and promoting the release of an anti-inflammatory cytokine (IL-10). DNPs have the potential to treat inflammatory injury.

## Figures and Tables

**Table 1:** Independent factor levels in BBD.

Level	Independent Factors		
	X_1_ (Liquid–Solid Ratio)/mL·g^−1^	X_2_ (Ultrasonic Times)/min	X_3_ (Ultrasonic Power)/W
−1	30	20	300
0	40	30	400
1	50	40	500

**Table 2:** Experimental design and results of BBD.

Run	Factors			Extraction Yield/%
	X1 (Liquid–Solid Ratio)/(mL·g−1)	X2 (Ultrasonic Time)/min	X3 (Ultrasonic Power)/W	
1	30	20	400	3.49
2	50	20	400	4.63
3	30	40	400	3.62
4	50	40	400	3.22
5	30	30	300	3.16
6	50	30	300	3.33
7	30	30	500	3.00
8	50	30	500	3.63
9	40	20	300	4.06
10	40	40	300	3.91
11	40	20	500	4.41
12	40	40	500	3.48
13	40	30	400	5.07
14	40	30	400	5.05
15	40	30	400	5.24
16	40	30	400	5.54
17	40	30	400	5.58

**Table 3:** Analysis of variance (ANOVA) results for BBD.

Source	Sum of Squares	Df	Mean Square	F-Value	*p*-Value
Model	0.0012	9	0.0001	32.29	<0.0001 **
X_1_-Liquid–Solid Ratio	0.0000	1	0.0000	7.10	0.0322 *
X_2_-Ultrasonic Time	0.0001	1	0.0001	16.80	0.0046 **
X_3_-Ultrasonic Power	2.720 × 10^−8^	1	2.720 × 10^−6^	0.0065	0.9378
X_1_X_2_	0.0001	1	0.0001	14.33	0.0068 **
X_1_X_3_	5.137 × 10^−6^	1	5.137 × 10^−6^	1.24	0.3029
X_2_X_3_	0.0000	1	0.0000	3.66	0.0973
X_1_^2^	0.0005	1	0.0005	126.06	<0.0001 **
X_2_^2^	0.0001	1	0.0001	18.62	0.0035 **
X_3_^2^	0.0003	1	0.0003	80.48	<0.0001 **
Residual	0.0000	7	4.156 × 10^−6^		
Lack of Fit	1.172 × 10^−6^	3	3.906 × 10^−7^	0.056	0.9803
Pure Error	0.0000	4	6.980 × 10^−6^		
Cor Total	0.0012	16			
R^2^	0.9765				
Adj-R^2^	0.9462				
C. V. %	4.92				
Std. Dev	0.002039				

*: 0.01< *p* < 0.05; **: *p* < 0.01.

## Data Availability

Data are contained within the article.
